# The Hospital. Nursing Section

**Published:** 1906-02-10

**Authors:** 


### The Hospital.

### Nursing Section.

Contributions for "THE HOSPITAL," should be addressed to the EDITOR, "THE HOSPITAL" NURSING SECTION, 28 & 29 Southampton Street, Strand, London, W.C.

No. 1,011.—VOL. XXXIX. SATURDAY, FEBRUARY 10, 1906.

### Notes on News from the Nursing World.

### THE ROYAL RED CROSS.

THE King has been pleased to confer the decoration of the Royal Red Cross upon Mrs. Eleanor Mary Hatch, in recognition of her services in nursing and attending the persons injured in the earthquake which occurred at Dharmsala last April. It may be remembered that in the account of a nurse's experience of the earthquake, which appeared in our issue of April 29, the sensation caused by the calamity was graphically described. But the more serious aspect was the personal suffering it was the means of inflicting, and we are rejoiced that His Majesty has recognised the services rendered by Mrs. Hatch by conferring upon her the high honour of the Order of the Royal Red Cross.

### LADY GREY AND THE NURSING MOVEMENT.

THE late Lady Grey—whose tragic death has provoked expressions of sympathy for her bereaved husband, the Foreign Secretary, all over the country, from the King down to the humblest workman on Tyneside—was warmly interested in the nursing movement. She was the founder of the Beadnell Nursing Association, under whose auspices the cottagers in the district can command the services of a village nurse in case of sickness; and she identified herself with various organisations in the North of England. Her active support could always be relied upon in respect to any local effort for the benefit of the sick and suffering.

### HOSPITAL NURSING AT MILAN.

AN English nurse who visited the Ospedale Maggiore at Milan—where it was stated the other day that some very favourable results have been obtained from experiments in the cure of tuberculosis of the bones and intestines by means of serum—sends us an interesting account of a visit which she recently paid to the large hospital. There were many features which she was not permitted to see, such as the Nurses' Home, or the building which does duty for it, and the laundry, from which one of the religious sisters in charge rigorously excluded her. The crowded condition of the wards which she reports, the lack of pretty ward furniture or decorations, the rusty surgical needles, the dirty scissors, the blood-stained mackintosh, the slovenly appearance of the nurses, and the small fumigating room for a hospital with 2,800 beds, indicate the vital importance of the developments which have already been partially finished. For some of these, even under archaic conditions, there is no excuse. Nothing can be pleaded in extenuation of dirty wards or slovenly dressed nurses.

### SUICIDE AT A DUBLIN HOSPITAL.

A MOST painful sensation has been caused at the Adelaide Hospital, Dublin, by the suicide of one of the nurses on night duty. The unfortunate young woman, who was thirty years old, was heard calling for help on Sunday morning soon after her retirement to rest, and upon another member of the staff entering her bedroom she exclaimed that she had taken poison in the form of two ounces of oxalic acid. In spite of immediate medical treatment she died in a short time. At the inquest on the following day it was mentioned that she had complained of severe headaches for some time and had been in very low spirits. The jury returned a verdict of death while temporarily insane, and added a rider signed by ten out of twelve, that the hours of work for night nurses at the Adelaide Hospital were excessive. This was in consequence of a witness stating that the deceased was in the wards from nine o'clock at night until nine in the morning. A late nurse, who tells us in a private letter that the nurses at the Adelaide are better off than in other hospitals, because accident cases are not taken in, writes in defence of the twelve hours of work. But we agree with the jury in thinking them excessive.

### HOSPITAL NURSES AND MARRIAGE.

A FRENCH physician, who has appealed in one of the French newspapers to ladies of good family in his own country to admire and imitate their English sisters who make hospital nursing their profession, has been assured that his appeal will not be responded to unless or until the woman worker of good family in France ceases to find it difficult to get married. We certainly do not think that hospital nursing is usually undertaken in England by educated young women because it is regarded as a door to marriage. There may be exceptions, but the girl who becomes a nurse either in this country, because she regards the hospital in the light of a marriage market, is, we hope, quite a rare exception. Moreover, judging by the comparatively small number of nurses who marry after they have completed their training, we doubt whether the work would be worth taking up for the ulterior purpose. At the same time, we consider the idea that a woman who takes to nursing as an occupation lowers herself in the social scale, to be very ridiculous. Nursing the sick in the right spirit can only prove elevating to character; and perhaps in due time our neighbours on the other side of the Channel will share our opinion.

### THROUGH THE GLASSES OF PREJUDICE.

In the current number of the *Lady's Weekly* there is an article headed "Does Professional Nursing Harden Women?" in which the writer, answering the question in the affirmative, asserts that a male friend "whose experience is as wide as his observation was keen" had come to the determination that it is far better when sick to be nursed by one's own womenkind, who, if they lack much in skill and scientific knowledge, make ample amends by their tenderness and infinite patience," than by a trained nurse. The writer recalls the case of a friend who was nursed by a Scotch "sensible" nurse who took away all her pretty beribboned nightgowns, dressed her in "good grey flannel" and made her life miserable so that she now speaks of hospital nurses "with a shivering distaste"; quotes the nurse who insists upon "having her regulated hours for sleep and going out though the heavens fall or the patient is in extremity," and keeps friends out of her patient's room; and affirms that though "men object to professional nurses," they have far less reason to complain of their ministrations than their own sex, for "few nurses hide the fact that they dislike to have women patients." The final conclusion is that "Because of the difference in their nature, paid men make good doctors, paid women unsatisfactory nurses." We can only say that the author has evidently good health and has no knowledge of the numberless kind-hearted, helpful, self-forgetful women who have in many homes, rich as well as poor, caused the name of "nurse" and "comforter" to be synonymous.

### OFF-DUTY HOURS AT CAMBERWELL INFIRMARY.

We understand that the night nurses and the day nurses at Camberwell Poor-law Infirmary have sent in a petition to the Guardians, the former asking for one night and the latter for one day off duty each month. At present the night nurses have from 1 until 10.30 p.m. off duty once a week, and every other Sunday from 1 till 9.30 p.m.; the day nurses having about the same time free. Nurses who have been in the Infirmary for twelve months have in most cases had one extra night off during that period. Camberwell Infirmary is a well-managed and up-to-date institution, but we think that the request of the nurses is very reasonable. Some of the work is extremely heavy, because there are so many old people—one is 103—and many paralytic and epileptic patients.

### GUARDIANS AND MATRON

At the meeting of Southwark Guardians last week, the advisability of advertising every vacant office on the nursing staff was discussed; but the question at issue appears to have been whether the candidates should appear before the Guardians in person, or should be selected by the matron of the infirmary. One of the past chairmen strongly supported the latter arrangement, which has been in force for some time, on the ground that there is now in the Infirmary "the finest staff to be found in any institution," whereas the old system, he contended, was bad, "because when the nurses had to appear before the Guardians for selection and appointment, the most pleasant-looking candidates were invariably chosen without regard to fitness." We do not know how far this indictment is true, but there is no doubt whatever that under the auspices of the present matron, Southwark Infirmary has become one of the best Poor-law training schools in England. The Guardians ultimately decided that the Committee should open all applications and then refer them to the matron for report. We can see no reason why the applications should not be addressed to her in the first instance.

### A NURSE'S SUCCESSFUL ACTION.

At the Clerkenwell County Court last week, Miss Lucy Ridsdale, a certificated nurse, sued the North Metropolitan Tramways Company for damages in respect of personal injuries and loss sustained. This was the second hearing. At the first the jury failed to agree and were discharged by Judge Edge. The plaintiff's case was that while alighting from a car belonging to the defendants, it re-started, throwing her upon her right knee and dragging her for two or three yards. She was unable to follow her profession for several months. The defence was that the plaintiff endeavoured to alight before the car stopped, but the jury found in her favour, with damages £75. We congratulate Miss Ridsdale upon her success.

### THE NURSE AND THE IRISH WARDMAID.

An inquiry has recently been held by the Irish Local Government Board Inspector of Workhouses into the suspension of a wardmaid at Glenties Union Workhouse, on the complaint of a nurse. The medical officer of the Union stated that he had been obliged to suspend the wardmaid upon two occasions for disobedience, notably because when attached to the scarlet fever ward she insisted on going into the main building. The nurse had asserted that the wardmaid had used abusive language towards her, and impeded her in many ways in the management of the hospital, refusing to boil, after washing, the clothes belonging to the patients. Nurse Owens, who was trained for four years in the Cork City Fever Hospital, gave evidence that at times the wardmaid declined to clean out the nurses' apartments, and stated that when the nurse did so herself the wardmaid threw the dirt swept up back again. She also surreptitiously gave a patient nuts, biscuits, and sweets. Upon examination later the wardmaid herself admitted that she went to the main building against orders, and that she opened a window in the ward which had been closed on the doctor's instance because "she thought it was necessary for ventilation." The proceedings closed with a vote of thanks to the inspector, whose report is awaited with interest.

### THE IMPERIAL MILITARY NURSING SERVICE.

We are officially informed that as Miss M. G. Fisher and Miss A. E. Harvey have been appointed staff nurses in Queen Alexandra's Imperial Military Nursing Service. Miss J. E. Dods and Miss L. E. Mackay, sisters, have been transferred from Queen

Alexandra's Military Hospital, Millbank, S.W., to the Military Hospital at Gosport; Miss A. Fitz-Gerald, sister, has been transferred from Cambridge Hospital, Aldershot, to Egypt. Miss H. McCurdy and Miss S. W. Tulloh, R.R.C., sisters, have been promoted to be matrons; and Miss M. B. Williams has been appointed staff nurse.

### MILITARY FUNERAL FOR A NURSE.

A great posthumous honour was paid to a nurse at Aldershot on Tuesday, when the remains of Miss Fitzgerald, a nursing sister who died of pneumonia a few days ago, were laid to rest with full military honours. The coffin was draped with the Union Jack, and many of the nurses at the Military Hospital, as well as several officers of the Royal Army Medical Corps, were present at the graveside.

### THE NURSES' CO-OPERATION.

The ordinary general meeting of the Nurses' Co-operation was held at 8 New Cavendish Street on Wednesday afternoon. The business was not of a controversial character, the principal event being the adoption of the fifteenth annual report and balance-sheet. According to the former, there are 493 nurses on the general staff, and 21 asylum-trained nurses who take mental cases only. The Co-operation have added to the 5 per cent. list all nurses who joined during the year 1900. It is stated that the work of the officers has been carried on during the past year in a very efficient manner, and the Committee congratulate the nurses on the results of the able and pleasant way in which their affairs have been administered by Mrs. Lucas and her staff. The excellent management of Miss Baker the sister in charge of the nurses' home, is also recognised. Mention is made of the death of two nurses during the year. In the finance report the receipts show an increase of £747 compared with the year 1904, but it is very properly pointed out that in 1904 there was a falling-off of £319 as compared with 1903. The amount paid to nurses was £795 in excess of 1904. The income of the society derived from commission fees earned by the nurses was £2,845, and it is in excess of all charges to the extent of £830. With the amounts added last year, the amount now invested in the name of the Co-operation is close upon £3,000; and, as at the end of last year the cash balance was £954, there ought soon to be an addition to the investments.

### AN UNUSUAL MODE OF MEETING EXPENSES.

In a new fever hospital in Lancashire, so recently erected that no wall has been built around it, and the garden is only separated from the surrounding fields by barbed wire, one day last month about thirty convalescent children were out enjoying the sunshine under charge of a nurse. Suddenly, the baying of hounds was borne on the wind, followed by the galloping of many horses. The hospital being situated in a hunting county, the nurse at once divined the cause of the strange sounds, and fearing that her charges might be in danger, she gathered them all into the laundry, the nearest refuge. A second or two later the poor little hare dropped into the garden, the hounds squeezed themselves through the barbed wire in mad pursuit over the flower beds and through the matron's precious rose-trees, and captured the terrified animal right under the matron's office window. In a few minutes there was nothing more to be seen save a bunch of grey and white fur! But good came out of the death of that hare, for the members of the hunt paid handsomely for the mischief they had wrought, and the matron found less difficulty than she had anticipated in meeting the extra expenses entailed by Christmas festivities.

### BAZAAR AT TORQUAY.

A bazaar in aid of the Torbay Hospital was held in the Bath Saloons, Torquay, on Wednesday and Thursday last week, when over £1,000 was realised for the benefit of the institution. The opening ceremony was performed by the Mayor and Mayoress of Torquay, in the presence of a large and representative gathering. The matron, sisters, and nurses of the hospital, in indoor uniform, were in charge of the Guild stall of plain needlework and other articles, the nurses going in relays as they could be spared from the wards, and a brisk trade was done.

### THE MATERNITY NURSE AND THE RAILWAY COMPANY.

At Manchester Assizes on Monday, Miss Tamar Manning, a maternity nurse, brought an action against the London and North Western Railway Company to recover damages for injuries sustained at Bramhall Station, on the ground of alleged negligence. The suggested negligence was that the nurse in getting out of the train fell and injured her thigh, the fall being due to the long drop from the carriage step to the platform. The jury, however, awarded a verdict for the railway company, though they strongly recommended that the platform should be altered. Miss Manning's only consolation is that her sufferings may ultimately result in the improvement of Bramhall Station, unless, a stay of execution having since been granted by the judge, she has the good fortune to succeed on appeal.

### SHORT ITEMS.

The Queen has graciously consented to become patroness of the Berkshire Nursing Association.—The post-graduate lectures which were given last year under the auspices of the Gateshead Nursing Association are being repeated this winter and are much enjoyed by the nurses. Next year it is expected that two courses will be arranged, one before, and one after Christmas.—The practice of seeking to increase the funds of the Ashton Sick Nursing Association by means of public begging in the streets is to be abandoned. After a recent collection the boxes were found to contain principally old tram-tickets, hairpins, and spent matches, the total amount in money scarcely exceeding twenty shillings.—At Chester-le-Street, on Tuesday night last week, Mrs. Watson, a well-known nurse in the district, was seized with paralysis when she was atending the Volunteer Ball, and died on Thursday without having recovered consciousness or speech.

### The Nursing Outlook.

"From magnanimity, all fears above;
From nobler recompense, above applause,
Which owes to man's short outlook all its charm."

### OVERWORKED PROBATIONERS AND NURSES.

It is sometimes helpful to the conscientious head of a large staff of working women to have the results of the system pursued brought to the notice of the employer by its effects upon the workers being driven home through the experiences of an outside observer. The Rev. J. B. Mackenzie, writing to the *Glasgow Herald*, gives his experience over long years of observation of the effect of the present system of training nurses upon many young women in his extensive country parish, Kenmore. Mr. Mackenzie points out that the women in question have learnt to live sparsely in a healthy mountainous district, where every one has to work hard in the open air, and so a healthy and energetic race is reared. A large number of women from Mr. Mackenzie's parish have taken to nursing, and they have been on the whole above the average both in physique and energy, whilst they have not entered on their training until they have attained an age considerably over that at which many women often make a start in other occupations in life. Despite these advantages Mr. Mackenzie's experience is, that the young women, who select nursing as a calling, break down in health in far larger proportions than any of their sisters, who obtain their living by other employments. Even those who avoid actual breakdown lose much of their old energy and buoyancy of spirits, owing to the amount of really hard work they have to do in the hospitals, coupled with the anxiety and strain of the exceedingly long hours they are kept on duty. Mr. Mackenzie contends that this weakening influence is increased by the circumstance that the probationers have to find the time necessary for study for their examinations, outside duty hours. Further, that they do not in fact have sufficient time for rest, whilst there is absolutely none left for "those innocent amusements which are necessary to refresh the mind after the strain of following with diligence an arduous occupation." This is a serious indictment, especially if Mr. Mackenzie's may be taken to represent the average experience of the clergy in country parishes.

More than once an attempt has been made to introduce an eight hours' day for nurses in institutions, but the expense of this change and other causes have so far defeated these efforts at reform. In some of the large provincial hospitals each nurse has to work on an average twelve hours per day, for where the time is reduced to 11½ hours on some days it is increased to 12½ on others, so that the average is never less than twelve hours' work out of each twenty-four. This state of things is still unfortunately too common in hospitals and kindred institutions, where nurses are trained. Some years ago the agitation in favour of reducing the hours attained a considerable measure of success; but that success has been diminished or destroyed owing to the stiffening of the examinations and the consequent increase in the number of hours which probationers have necessarily to devote to study, if they are to pass their examinations and obtain their certificates. We believe that this fact has been largely overlooked by many in authority, and the whole question of the present hours of work of nurses of all grades in public institutions should be reconsidered on its merits, with a view to the introduction of early and substantial reforms.

It must be bad economy to overwork any member of the staff of a public institution. The older matrons used to have a theory that it was wise to put as much strain as possible upon a probationer in her early days, and to keep her standing about so as to test her physical powers to the utmost. We have never had any sympathy with these views, and have always held, that every matron and lady superintendent owes it to the credit of her hospital, and to her own reputation, that she shall treat each of her probationers considerately, and set herself to get from the staff the best work which individually and collectively they are able to give, by exercising the utmost care in supervision, so as to encourage and strengthen the least fit. For in this way alone, can she hope to raise to a maximum, the health and capacity of the whole staff entrusted to her care.

There must be something wrong in the system pursued at any institution where overwork and strain are permitted to cause a breakdown in health to young women of strength and energy, which their early life and surroundings have afforded them in a healthy mountainous district like that of Kenmore. We would venture to suggest that at least one member of each hospital committee should make it his business to take up this question of the working hours of the probationers and nurses, and to inquire into the facts at his institution. Such a step would be welcomed by the more intelligent of his colleagues, at any rate. No gentleman can care to be associated with the management of any institution, which directly or indirectly does injustice or injury to the youngest and most defenceless members of its working staff. Let such an inquiry therefore be systematically instituted at every hospital and institution employing nurses. Where there are no such abuses as Mr. Mackenzie reports, let the facts be published. Where they are found to exist let a remedy be found and applied with all possible speed. Our columns are freely open to such reports and facts.

### The Care and Nursing of the Insane.

By Percy J. Baily, M.B., C.M.Edin., Medical Superintendent of Hanwell Asylum.

### I.—ANATOMY AND PHYSIOLOGY.

(*Continued from page* 259.)

3. *Joints.*—A joint is formed by the contact of two or more bones. As a rule the object of a joint is to allow of motion. The amount of motion which may occur at any joint is very variable, and in some there is none. Hence joints are classified into three main classes:—(1) Those which are immovable; (2) those which are partly movable; (3) those which are freely movable.

1. Immovable joints are such as occur between the bones of the head and face. Frequently the edges of the bones which come into contact with one another are serrated or toothed, the teeth of one bone fitting tightly into the notches of the other. The two bones are separated from one another only by the periosteum, and are firmly tied together by ligaments.

2. Partially movable joints are found between the bodies of the vertebræ. The opposing surfaces of bone are separated from one another by a thick pad of cartilage, and are bound together by strong ligaments. The amount of movement which occurs at these joints is appreciable but very limited.

3. Movable joints are all constructed on the same plan. The surfaces of the bones which come into contact with one another (articular surfaces) are covered with a layer of cartilage, and the joint is surrounded by a strong fibrous capsule which is thickened in parts to form bands or ligaments. These ligaments are (generally speaking) attached to the bones beyond and around the cartilage which covers their articular surfaces, and are sufficiently loose to allow of the requisite amount of motion. The inside of the joint is lined by a delicate membrane, which however, does not extend over the cartilage; it secretes a thick syrupy fluid which acts as a lubricant to the joint. The membrane is called the synovial membrane, and the fluid it secretes is the synovial fluid. It is this structure in the joints which becomes inflamed in rheumatic fever. The muscles which surround a joint help to strengthen it and to keep the bones in their proper places. Movable joints are found in a variety of forms, which allow of various kinds of motion. Of these the most important are:—

1. Gliding joints. In these the articular surfaces are nearly flat, and the only kind of motion possible is one in which one surface glides over the other. Such joints are found between the small bones of the wrist and foot.

2. Hinge joints in which the kind of movement resembles that of a hinge—that is to say, movement at these joints is only possible through one dimension in space. Examples of such joints are the knee and elbow.

3. Ball and socket joints in which the rounded ball-like end of one bone fits into a hollow cup in the other. In these joints movement occurs through all dimensions of space. The shoulder and hip-joints are examples.

Certain terms which are applied to the various kinds of movement which may take place at any joint require explanation. When a limb is bent upon itself, as when the hand is brought towards the shoulder by bending the elbow or when the foot is raised towards the buttock by bending the knee, the movement is known as flexion. The opposite movement, that of straightening the limb, is called extension. When a limb is carried away from the middle line of the body, as when the arm is carried out from the side or when, standing upon one foot, the other is carried out sideways, the movement is known as abduction. The opposite movement is adduction. The terms pronation (lying upon the face) and supination (lying upon the back) refer to the movement of the radius around the ulna. If, when sitting at a table, the hand is allowed to rest upon it with the palm upwards the hand is said to be in the supine position, the radius is then lying along the outer side of the ulna. When the hand is turned over so as to lie upon the palm it is said to be in the prone position, and the movement from the one position to the other is called pronation. During this movement the lower end of the radius rotates around the lower end of the ulna and carries the wrist and hand with it. In the position of pronation the radius lies more or less across the ulna. The change back again from the prone to the supine position is effected by the movement of supination. Circumduction is the name given to the movement which occurs when the arm is allowed to swing round and round at the shoulder joint, or when, standing upon the toes of one foot, the other foot is made to perform a circular movement from the hip-joint. "Rotation signifies movement of a bone round its axis without any great change of situation." If the arm is allowed to hang at the side of the body with the palm looking backwards, it can easily be turned completely round so that the palm looks forwards. Much of this movement occurs below the elbow-joint, and is one of supination, but if the arm be grasped by the opposite hand while the movement is being made the arm bone can be felt moving. This movement of the humerus is one of rotation at the shoulder-joint.

### 4. The Muscular System.

The muscles are the agents of motion. The peculiar property of muscular tissue is its ability to alter its form. This power of contraction, as it is called, is brought into action at the bidding of the nervous system, as we shall see later. Every movement which is performed with or within our bodies depends upon muscular contractility.

There are two varieties of muscles. One variety is under the power of the will and acts only in response to the wishes of the individual. Hence this sort of muscles is spoken of as voluntary muscles. The other variety, although equally controlled by the nervous system, acts entirely independently of the will, and these muscles are therefore called involuntary muscles.

(1) *The Voluntary Muscles.*—These compose the greater portion of the bulk or flesh of the body and constitute the lean part of a joint of meat. As an organ, a voluntary muscle consists of more than mere muscular tissue. It is composed of many bundles of muscle fibres which are enclosed within the meshes of a network of fibrous tissue in which run many blood-vessels and nerves. These meshes or partitions are continuous with a fibrous covering or sheath, which surrounds the whole structure. At one end (sometimes both) this fibrous tissue is generally prolonged beyond the muscle-tissue and forms a strong cord-like band called the tendon or leader. The fleshy portion of the muscle is called the belly. Most of the voluntary muscles are attached by both their ends to different bones, consequently, when the muscle shortens or contracts, the bones are moved. The tendon is usually attached to the bone which undergoes the greater motion (the insertion of the muscle). The attachment of the fleshy end or belly of the muscle (which occupies a much greater surface of bone than the insertion) is to the more fixed bone (the origin of the muscle). A few of the voluntary muscles—those of expression—are attached to the skin of the face and by their action alter the appearance of the features in response to various emotional states.

(2) *The Involuntary Muscles.*—These are found in the heart, in the walls of the blood-vessels, in the walls of the stomach and intestines and of the bladder, etc. They have no tendons and exist generally as thin layers of muscular tissue (except in the heart, where they occur in considerable bulk). They cannot be affected by the will although they are controlled by the nervous system and are easily influenced by certain emotional conditions, as is seen in the act of blushing. They are concerned in such vital actions as the circulation of the blood, digestion, etc.

*(To be continued.)*

### The Nurses' Clinic.

### PREPARATIONS FOR MINOR OPERATIONS IN A PRIVATE HOUSE.

It often becomes the duty of a nurse to prepare for a minor operation in a private house, and as she must make all her preparations before the arrival of the surgeon and anæsthetist, it will be necessary to be at the patient's house at least an hour before the time arranged for the operation. The question of a suitable room is generally settled by the doctor, who will be sure to choose a room with a good light; all other details are usually left to the nurse. The first essential in the temporary operation room is a suitable operating table. This should be as near as possible of the dimensions used in an operating theatre. Very often a kitchen table being long and narrow approaches nearest to the ideal. If the patient is a baby or young child a chest of drawers of moderate dimensions covered with a folded blanket will make an excellent operating table. Place upon the blanket a sheet large enough to tuck in all round, then a piece of mackintosh sheeting (if the house possesses one) in the neighbourhood of the part to be operated on and over this a draw-sheet. A firm pillow for the patient's head, and two or three blankets to cover him completes this part of the preparations. It is wise to have a small bright fire burning unless the weather is sultry. Two or three small tables covered with white cloths for the reception of lotion bowls and instrument dishes must be in readiness. It will be wise to have at hand one or two shallow pie-dishes, though the surgeon may bring his own dishes for instruments. One can of boiling water and another of cold boiled water, bowls for lotions, wash-hand basins for the doctors, a bucket to receive soiled water, soap, nail-brushes, and a supply of towels must all be provided. The dressings will be provided by the surgeon, but the nurse should have safety-pins, surgical scissors, and tongue-depressor at hand. Two small receivers, one for soiled swabs and one for the use of the patient, in case of vomiting, will be necessary. The bed must be prepared by placing towels over the pillow, and some protection over the part where discharge or bleeding is likely to occur. Hot-water bottles covered with flannel should be in the bed before the operation takes place. The nurse will be wise if she removes all superfluous light articles from the room, such as garments hanging behind doors, etc., before the doctors arrive. She should also inquire whether the bowels have been recently relieved, and at what hour the last meal has been taken. The patient must be clothed lightly but warmly, and if a woman the hair should be parted in the middle and arranged in two plaits, and false teeth removed from the mouth. In no case should the patient see what preparations are being made for his or her benefit. If he must be in the room, let a screen be placed around the bed, so that he is not made nervous nor unnecessarily excited by the nurse's movements and arrangements. In the case of a young child a towel stretched across the chest and fixed with safety-pins on either side will be a useful precaution and probably give more freedom to the surgeon during the operation. A nurse single-handed can manage very well if she has thought of every detail beforehand, and is ready to give intelligent assistance during the operation, and having once discovered the arrangements which satisfy the surgeon she will always know what to prepare for him in the future. For major operations, of course, more elaborate preparations are necessary, but if the nurse has prepared all the above-mentioned details with the addition of large towels which can be made to do duty as doctor's aprons, she may make her mind easy and calmly await the course of events.

### To Nurses.

We invite contributions from any of our readers, and shall be glad to pay for "Notes on News from the Nursing World," "Incidents in a Nurse's Life," or for articles describing nursing experiences at home or abroad dealing with any nursing question from an original point of view, according to length. The minimum payment is 5s. Contributions on topical subjects are specially welcome. Notices of appointments, letters, entertainments, presentations, and deaths are not paid for, but we are always glad to receive them. All rejected manuscripts are returned in due course, and all payments for manuscripts used are made as early as possible after the beginning of each quarter.

### Incidents in a Nurse's Life.

### TRAVELLING WITH A DIPSOMANIAC.

It recently fell to my lot to accompany a dipsomaniac from a nursing home in Kent to a boarding house in London, and never shall I forget the trouble and annoyance she caused me. We were escorted to the station by another member of the nursing staff, and comfortably settled in a first-class reserved compartment; but no sooner had the train started than my worries began. First of all the patient made a pretence of feeling faint, having found out that I had a flask of brandy in my dressing bag; but, finding I was firm as adamant in refusing her, she successively shrieked, wept, and moaned, threatening to do desperate things. Seeing that I would not relent my patient gradually calmed down and sat sulkily in her corner for the rest of the time. But the worst was yet to come. I knew we should not be met at our journey's end, so, while the train was slackening speed, I was turning over in my mind how best to see after the luggage, have our little dog released from the guard's van, and yet hold on to my patient! The difficulty was solved by the patient herself, who, as soon as the train stopped, jumped out of the carriage and, taking to her heels, steered a straight course to the haven of her desire —to wit, the refreshment room. After a few hurried directions to a porter, and with the dog at my heels, I followed in her wake, arriving in time to prevent any stimulant being taken. Needless to say, my patient was furious, and, as she began to make a disturbance in the station, I was obliged to call to my aid a police officer. He kindly but firmly placed her in a cab, telling her that if she gave me any further trouble he himself would relieve me of all responsibility. This had a wonderful effect, for she sat quietly in the cab, whilst the luggage was arranged upon it. We drove off, but had not travelled far before I was treated to a second edition of the moans, groans, and tears, which had been used to soften my heart in the train. These lasted until we arrived at our destination. Here the patient resolutely refused to alight, and in consequence soon attracted quite a large crowd. Picture to yourself this scene! Cabby seated on his box, not caring in the least how long he sat there; two runners, hot and exhausted from their efforts to keep up with the cab (for I had been too weary to do more than expostulate with them on the way), the dog barking with all his might, the crowd wondering what monster was going to emerge from the vehicle, and last, but not least, the landlady herself, most indignant that such a commotion should take place in front of her genteel and highly respectable establishment.

"'Tis a long lane that has no turning," as it proved in this case; for, after much persuasion and many threats, my obstreperous patient condescended to go quietly into the house, where the little dog, tired out with the unwonted excitement, had preceded us and made himself quite at home. I then settled up with cabby and the "runners" and followed my patient. She had quite changed her tactics, and was full of remorse, falling on her knees and imploring my pardon for her disgraceful behaviour.

When I eventually said "good-bye" it was with a thorough determination never again to undertake the care of a dipsomaniac. It was my first experience of the kind; but, alas! it has not been my last, as I am continually meeting with similar cases in every grade of society I begin sadly to believe in the statement often made: once a woman has given way to intemperance, it is almost a hopeless task to try and reclaim her, and I feel sure that many of my fellow-nurses could echo my sentiments.

### A Matron's Responsibility to her Probationers.

BY ONE WHO HAS BEEN BOTH.

In hospital life it should be an understood thing that the patients and their comfort must come first, but surely the training we can give our probationers, and the variety of experience we can provide to fit them for their future career, must come immediately after. It is not always the simplest matter to keep the probationers steadily working on in one ward for a stated period of two or three months and then move them on to just the most suitable variety of work, when for many reasons it would be more convenient to move them more frequently and with less regard to what they need to learn. For instance, a probationer in the children's ward develops measles. You at once think Nurse A. will be back from her holiday to-night; she must take her place. But on second thoughts you remember that Nurse A. was night nurse in the children's ward not long ago, and she has had no men's medical training. Is there a nurse from the men's medical who can go to the children's ward? No; the one who has been there long enough to be moved is too junior to send to the children, but she might go to the women's surgical, and one from there to the children, and thus the nurse returning from her holiday can go to the men's medical, and the vacancy in the children's ward will be filled by a suitable probationer. But all this takes a little time to think out, and is only one of the many problems that have to be worked out at frequent intervals.

I think that for the first two years it is most essential not to move the probationers more frequently than every two or three months when it is at all possible, and if this is done regularly then in the third year they will be much more capable of grasping the work when they have to be changed about more frequently.

In the hospital of which I am thinking just now—a provincial hospital of 50 beds—the wards are rather large, so that the medical and surgical cases have to be mixed, but there still remains a good variety of work for the probationers, as there is the children's ward, the private wards, the theatre, and the district. I always tried to arrange for the probationers to go on night duty for three months at a time, but never longer. Lengthy spells of night work are very trying to the health of young nurses. Two married ladies whom I have met lately and who are still in feeble health, though their training is now in the past, attribute their weakness almost entirely to the overstrain which they underwent at one of the largest hospitals in the North of England, where it was the custom—and I believe it still is—for the nurses to go on night duty for a whole year at a stretch. They look back on that twelve months' work in heavy wards as a truly "killing" time. When my probationers went on night duty they spent the first six weeks on the male floor (usually the lightest), and the second six weeks on the female floor, or in the children's ward. This three months was very rarely interrupted, except by illness. For the wards on day duty, the theatre, and the district two months was the usual thing without a move. The district probationer goes out every morning from 9 to 12.30 to assist the district nurse (who has a larger district than she can possibly get round alone), and most of the probationers would have two spells of two months each on the district in the course of their three years, and in the mornings and evenings help in the children's ward. This district training, under an experienced nurse, has proved a very valuable adjunct to the hospital course. My probationers often stayed on with me for another year after they had gained their three-years' certificate, and in this fourth year they were extremely useful to the hospital, taking the district nurse's holiday, the ward sister's and night sister's holiday, and also going out occasionally as private nurses. We had a small private nursing staff attached to the hospital, and as it was chiefly employed by doctors on the hospital staff, it was nice for these nurses to begin their experience of private work for doctors whom they already knew and understood. There was a fever hospital a few miles away, and the authorities there frequently sent to us for an extra nurse, and this, too, added to the experience we were able to give to nurses who stayed on for a fourth year.

I will add the plan which I always kept in my desk to remind me of what work the probationers had done and what they still required to do, and I found that a reference to this plan every few months would often check me when, for my own convenience, or for that of the ward sister, I wanted to move a probationer who should not be disturbed, or when I did not want to move a probationer who really ought to be moved on.

I am quite sure that it is order and regularity, combined with absolute justness to all, which makes life in hospital such a cheery, happy time; and when nurses recognise that they are treated as reasoning women they very rarely fail to give that loyal and hearty service that is so essential for the patients' comfort and for creating a happy atmosphere in the hospital.

FORM I.

| | |
|---|---|
| NURSE A. (2nd Year) | Sen. Pro. Female Floor, Jan. 1st to Feb. 29th. District Pro. March 1st to April 30th. Night Duty Male Floor, May 1st to June 14th. Night Duty Female Floor, June 15th to July 31st. Holiday, Aug. 1st to 21st. Special Private Ward, Aug. 22 to Sept. 3rd. Theatre Pro., Sept. 4th to Oct. 31st. Sen. Pro. Male Floor, Nov. 1st to Dec. 31st. |
| NURSE B. | ............................. |
| NURSE C. | ............................. |

(This form filled up each week from the daily diary; but, of course, the nurses cannot always be kept to the exact dates as in this sample.)

FORM II.

| | Day Duty. | | Night Duty. | | Special Nurse. | Theatre Probationer. | District Probationer. | Holiday. | Off duty, ill. | Total. |
|---|---|---|---|---|---|---|---|---|---|---|
| | Male Floor. | Female Floor. | Male Floor. | Female Floor. | | | | | | |
| | days | days | days | days | days | days | days | days | days | days |
| NURSE A......... (2nd Year.) | 61 | 60 | 46 | 47 | 14 | 55 | 61 | 21 | — | 365 |
| NURSE B. ...... (1st Year.) | 112 | 123 | 45 | — | — | 61 | — | 14 | 10 | 365 |
| NURSE C. ...... | — | — | — | — | — | — | — | — | — | — |
| NURSE D. ...... | — | — | — | — | — | — | — | — | — | — |

(This form filled up every six months, and any omissions in training kept in mind for the following six months.)

### The Ospedale Maggiore at Milan.

IMAGINE if you can a great building containing more than three times as many patients as the London or Bartholomew's, and you will have but a dim impression of the magnitude of the "Great Hospital" at Milan. It was founded by Duke Francesco Sporza and his wife, Bianca Maria, in 1456 on the site of an old palace. The architect was one Antonio Filarete, the Florentine who designed the great gates of St. Peter's. It is quite one of the grandest domestic buildings in Northern Italy, and it contains no fewer than nine courts. The older part is covered entirely with terra-cotta, and Street says that these terra-cotta enrichments can hardly be surpassed by any others of the same period in Italy. The hospital contains 2,800 beds, of which few seemed to be empty in the wards we went through. There were 20,500 patients admitted in 12 months, and 2,700 deaths; the mean mortality is about 13 per cent. We—a friend and myself—entered from the Via dell' Ospedale into the principal court of the hospital. This was designed by Richini in the 17th century, is surrounded by arcades, and has monuments to the most eminent medical teachers, under the porticoes of the quadrangle. We explained our errand, and an orderly was at once sent to show us all we wanted to see. He took us over the old hospital first, which, though it may be charming architecturally, is anything but charming hygienically.

### THE WOMEN'S MEDICAL WARD.

We started with the women's medical ward—a long room with 40 beds, so close to each other that patients lying in bed could easily touch the bed on either side of them. Long windows gave light down one side of the ward only, and two beds were placed between each window. Altogether 40 patients to nine windows! There was a small wooden locker between each bed, with a vessel (clean) arranged conspicuously on each. Curtained over at the bottom of the ward was a large space, about the use of which our guide was rather vague, but we gathered that it was for medical examinations and small dressings. The ward looked depressing, owing to the lack of books, plants, flowers, and (to our English eyes) necessary ward-furniture. But the patients looked happy and well cared for. The next ward was one reserved for tubercular cases. This contained 30 beds, and we were glad to see that the beds were further apart, the ward much better ventilated, and the vessels not *en évidence*. The bedding in every case seemed exceedingly clean. The patients in this hospital lie upon two mattresses, the bottom one filled with grass, the top one with wool. We were assured that the bedding and clothing of every tubercular patient in the hospital were invariably fumigated.

### THE SURGICAL WARD.

The next we visited was a women's surgical ward, which was certainly disappointing. There was not the same overcrowding as in the women's medical ward, and the ventilation was better; but we noticed surgical needles that were rusty, scissors which were anything but clean, and a sort of wooden operating-table with a clean sheet on top, and a dirty mackintosh with bloodstains on it underneath! In a sort of ante-room to this ward we saw a number of pairs of very large wooden, not very clean-looking, shoes. These were to keep the doctors' feet from getting wet during an operation! We went on from ward to ward, and the conditions seemed much the same: excessive overcrowding, as we should certainly deem it in England, in many of the wards surgical cleanliness seemed unknown, and the bathing arrangements, etc., distinctly primitive. The nurses—or *infermière*—looked untidy and rather slovenly. They are dressed in

### THE OSPEDALE MAGGIORE AT MILAN—*continued.*

blue and white-striped dresses, with no collar, apron, or cap. In many of the wards we saw a nun or Sister of Charity in charge of these *infermière*, but we could not ascertain whether there was one in each ward, nor how many there were altogether.

The Kitchen and Fumigating-room.

We passed rather gladly from these depressing wards to a most cheerful and clean kitchen, where we saw excellent soup and toothsome-looking vegetables being prepared for dinner. We tried hard to get into the laundry, but the nun in charge was obdurate and would have none of us. So we repaired to the fumigating-room, which contained absolutely nothing but a man and a large disinfector (Lyons pattern), neither of which was working. We were told that the clothes and bedding of every infectious and tubercular patient in the hospital were stoved here. This may have been so, but it seemed a very small place for such a purpose. We were only allowed to peep into the infectious ward, and saw a long row of little black cots. Here we were told that measles, chicken-pox, and scarlet-fever patients were admitted, but whether all mixed up together or not our guide did not reveal.

The New Hospital.

We then crossed the road, past the chapel (where there are some fine portraits of benefactors to the hospital), and the "skin" wards, which we did not see, through an enormous vegetable garden which supplies the whole hospital, and finally arrived at the new hospital, which is built in blocks or long pavilions. The wards here are quite charming, and as up-to-date as those in the old hospital are mediæval. The floors are of terazzo, the walls distempered, everything the acme of cleanliness and neatness. Later on, when all the building is finished, this new building is to be the surgical part of the hospital; then the old is to be renovated and converted into the medical part.

The Children's Surgical Ward.

We went first to the children's surgical ward, where there was a sweet-faced nun in charge, who was very proud of her pretty ward, and pleased to show it to us. The babies in bed wore pale blue; those up wore pink; and this, with the brightly-painted toys on each bed, made a very pretty bit of colour—a great contrast to the wards we had just seen. It was dinner-time, and peace and happiness reigned, except for one poor lady with a cerebral abscess, who moaned incessantly. Amongst other cases we noticed a little girl undergoing the new *excusiore* treatment for congenital hip. There was a small operating-theatre attached to this ward, and though perhaps it was not as elaborate as those we are accustomed to see in London, yet it was quite up-to-date, and as spick and span as it could be with glittering brasses, shining glass, and spotless instruments.

The Rest of the Building.

Parallel to this is the children's medical ward—always rather a sad ward, but as cheerful as possible, and presided over by the same kindly nun. The second block is being used just now as men's surgical wards, and the third block has been fitted up most elaborately as an electrical and pathological department, with every new appliance, a Röntgen and *x*-Ray room, a photographic department, hot-air baths, etc., etc. One room contained several mechanical contrivances for crooked spines, many of them quite archaic, and never used now—quite an interesting collection. We came out by a side door, and found ourselves by the "Nurses' Home" as we should call it—a large house where tne nuns and *infermière* live. Unfortunately we did not see it, as strangers are never allowed inside it.

### District Nursing and Diet.

### EXAMINATION QUESTIONS FOR NURSES.

The question was as follows: If called upon in district nursing or otherwise to arrange the diet of a person suffering from an exhausting disease, such as consumption, and if that person belonged to a labourer's family (consisting perhaps of six or seven people) whose wages did not exceed 18s. or thereabouts per week, what should you advise as the cheapest and most nourishing scale of diet?

First Prize.

For a patient suffering from consumption I should advise as much fatty food as possible, such as dripping, which can be used instead of butter and can be bought for 4d. or 5d. per lb.

Fat bacon boiled is very good, and the fat that rises can be clarified and used and the liquor used as a basis for soup, 2d. or 3d. of vegetables, a few pieces and bones, with either some lentils or peas and some suet dumplings will make a good dinner. Suet pudding is very nourishing and is good with currants, a few raisins or sultanas, or plain with the old-fashioned treacle.

Fresh herrings when in season are both cheap and good: they contain a good deal of fat; bloaters, too, are nourishing when in good condition.

Boiled dripping crust, if lightly made, is cheap and good. Bread made with seconds flour is best, and any stale pieces will make a good pudding if soaked in sweetened milk, and a few currants, sultanas, or raisins, and all beaten together and either boiled in a basin or baked.

A joint for a family of five or six would be out of the question, but a nice tasty dish could be made of some pieces that have been cut from other joints to trim them; could be stewed with onions, carrots, and turnips and potatoes.

Plain boiled rice with currants or raisins and sago, rice and tapioca pudding can be made with skimmed milk.

Porridge made with coarse oatmeal and bread and milk are both light and nourishing for supper.

I should advise cocoa to drink in preference to tea or coffee.

Hot dripping toast, if it can be digested, is good, and, with a cup of cocoa, would make a good breakfast, or bread and dripping would do instead if the toast is too rich.

Cheese is very nourishing, and should be eaten in the middle of the day.

Fresh haddock, hake, cod, and sprats can often be got cheap when there is a good season, and can be cooked in a variety of ways.

Wild rabbit baked in a little skimmed milk with a little onion makes a very good dish. Jam is cheap nowadays, and will often tempt the appetite, and can be used for puddings.

Potatoes should form one of the chief foods.—"Billy."

Second Prize.

The food must be well cooked and varied as much as possible, and not too long a time must elapse between each meal.

Bread made from coarse flour (bought from a miller) and baked at home would be cheaper and more nourishing. Skim-milk can be bought at many country places from the farmers at about one penny a quart, and could be used for cocoa, puddings, etc.

For breakfast I should advise porridge followed by dripping toast—fat pork or fat bacon toasted in front of the fire with bread beneath to catch the drippings—and cocoa. In the middle of the morning a glass of hot milk or cup of soup. Twopennyworth of bones well boiled will make quite a lot of good nourishing soup.

For dinner trimmings of meat can be bought at about fourpence a pound and can be made into puddings (minced), stewed potato pie, or baked in batter; the cheap parts of fat bacon and pork boiled (the liquor to be kept for pea-soup); tripe cooked with onions; sheep's head boiled: the broth is very good eaten with suet dumplings, and the brains could be boiled and eaten with bread and butter for supper; suet, rice, or bread puddings, any vegetables or fruit obtainable—rhubarb, blackberries, etc.

For supper any cheap fish, such as herrings when plentiful, dabs steamed or cooked in milk—heads of big fish and odd pieces can sometimes be bought very cheap at the time the shops close—soup or cocoa.—"Ashes."

The Cause of Feeble Answers.

Last month's question seems to present insurmountable difficulties to many, indeed almost all, candidates, though there is a most welcome improvement on the whole in the papers sent in this time compared to those received on the same subject more than two years ago. But now, as then, the majority of nurses seem to have very elementary ideas as to what 18s. will do in providing for a family. Meat more than twice in the week would not be possible, and I should like to know the locality where an entire sheep's head could be bought for fourpence. It would be well if all nurses would make a plan to acquire some practical knowledge as to the cost of different articles of food. "Billy" and "Ashes" are the prize winners, their answers being "all round" the best, though in some particulars the gainers of honourable mention beat them. "Billy" mentions cheese as a nourishing item of diet: this is true, and nurses do not seem to be sufficiently aware of it. Authorities tell us that the amount of nourishment in cheese is very large, whereas the less well informed have an idea that it is of very doubtful benefit and inclined to promote indigestion. "Ashes" would learn by experience that very seldom could fish be afforded for supper, if soup or meat had composed the mid-day meal.

Honourable Mention.

This is gained by "Alec," "R. T. R.," "Glasgow," "Swallow," and "Ireland." "Alec" sends an excellent answer, but, unfortunately, it was so carelessly written and so full of mistakes that publication was impossible. In fairness to others, no corrections can be made, and so this paper, which merited a prize in other respects, can only receive honourable mention. "R. T. R." also sends a good answer, and "Swallow" mentions an excellent old-fashioned food—only noticed by a few candidates—namely, boiled milk with finely chopped suet in it.

Question for February.

What would you do in a case of severe burn when the doctor lives several miles away, and may probably not arrive for many hours?

The Examiner.

### Presentations.

Southall-Norwood Sanatorium.—On Saturday afternoon last Miss Constance L. Williams, matron at the Southall-Norwood Sanatorium, gave a farewell tea to the staff on the occasion of her resignation. Miss Williams was the recipient of an afternoon tea service, presented by the nurses and domestic staff at the Sanatorium as a small token of the respect and esteem in which they hold her.

### Novelties for Nurses.

(By Our Shopping Correspondent.)

The Nurses' Outfitting Association, of Stockport, has just issued a new catalogue of nursing requisites, etc., which nurses would do well to procure and keep by them for reference, as the prices given are reasonable, and the firm has an excellent reputation for the quality of their goods.

### Royal British Nurses' Association.

ANIMATED MEETING.

A special general meeting of the Royal British Nurses' Association was held at 11 Chandos Street on Wednesday, when the room was crowded, many remaining standing throughout.

The chair was taken by Sir James Crichton-Browne, who was supported by Dr. Comyns Berkeley (Secretary), Dr. Bezley Thorne, Mr. Pickering Pick, Mrs. Coster, Dr. Clement Godson and others.

Dr. Comyns Berkeley read the notice of the meeting to the effect that at the special general meeting held on January 17 a resolution was moved by a person who, not being a member of the Association, was not entitled to take that course, and that as it was feared that other persons not being members might have taken part in the voting, the present meeting had been called. Several letters dissenting from the object of the meeting were read.

The Chairman then referred to the fact of a resolution at the meeting of January 17 being moved by a lady who was not at that time a member of the Association. (At this point there was considerable interruption and cries of "Name.") Sir James Crichton-Browne, however, went on to say that their legal advisers had assured them that under the circumstances the proceedings must be rescinded.

Dr. Comyns Berkeley moved the following resolution:—

That the resolutions passed at the special general meeting of the Royal British Nurses' Association, held on Wednesday, January 17, 1906, be rescinded, and that the draft Bill be re-introduced in the form in which it left the special meeting of the General Council held on Monday, January 8, 1906.

The resolution was seconded by Miss Kuys, and after some irrelevant discussion was adopted by a large majority.

The Nurses (Registration) Bill, as amended at the meeting of January 8, was then read, and passed clause by clause.

Upon section 11 of clause 4 there was a long and heated controversy. It was proposed by Miss Tawney, and seconded by Miss Campbell-Thompson, that the number of elected registered nurses should be increased to three.

Mrs. Bedford Fenwick proposed that the number should be increased to seven, and affirmed that the feeling of doctors in the West End was that the nurses should be adequately represented on the Board. She delivered a message from her husband to the effect that he trusted "that they would not disgrace themselves before the nurses of the world by not insisting upon an adequate representation."

In the course of the discussion that followed a supporter of the movement for additional representation of nurses remarked that she much regretted that Mrs. Bedford Fenwick should have spoken with so much heat, adding that some of the things she had said were scarcely in good taste. To which Mrs. Bedford Fenwick replied that "this was too serious a matter to consider taste."

Dr. Bezley Thorne, who was subject to constant interruption, pointed out that medical men would not consent to accept a subordinate position on the Board, as would inevitably be the case if they were in a minority.

Miss Tawney's amendment was then put to the vote and carried, Mrs. Bedford Fenwick's amendment being lost.

The whole of the proceedings were characterised by unusual demonstrations of feeling, which Sir James Crichton-Browne, as well as Dr. Bezley Thorne, found it necessary to deprecate. Amongst the more prominent interrupters were Mrs. Bedford Fenwick and Nurse Beattie.

### Everybody's Opinion.

### NIGHT NURSES' HOURS.

"A. M. M. B." writes : I see in Tuesday's paper that a Dublin jury, in giving a verdict, say that they consider the hours from 9 P.M. to 9 A.M. too long for night nurses. Now, as a matter of fact night nurses on an average have far lighter work than day nurses; also if there are any particular, trying, or difficult, or burnt cases, help is always given to them. There is also a night sister, who comes in several times during the night, and a great part of their work is simply to watch sleeping patients or listen for calls. They can often study and sew, and much improvement can be made in one's studies when on night duty. Another point is that, although a night nurse is on duty from 9 till 9, yet the first three hours there is hardly anything to do, and the last three the day nurses are buzzing round; and when once the breakfasts are in, if a woman is anything like a good manager, and not one of the proverbial grumblers to be found in all walks of life, she will find that night duty has—at least for the more than average nurse—no fault as regards the hours, and that all hospitals must arrange duty hours, not for the exceptional, but for the happy, ordinary, level-minded person. Surely this sad case in no way called for such a sweeping rider, which can be applied to almost all our hospitals.

### BLUFF AND BLUSTER.

NURSE LUNN writes : I hailed with delight your article on bluff and bluster in THE HOSPITAL. It has made me boil with indignation to see the spirit of arrogance and self-assertion which is being fostered in the minds and lives of trained nurses by those who "think that they are doing good service" by the fight for nurse representatives at the General Council. It may be all right for nurses in hospital to take the high hand; but it will be the ruin of private nurses. Our living depends entirely upon the recommendations of medical men. Are they likely to employ nurses in whom the spirit of rivalry and insubordination has been encouraged? I have been a private nurse for seven years, and can testify to the kindness, courtesy, and consideration with which I have been treated by every doctor under whom I have worked. I am sure that there are hundreds of others who would willingly entrust their welfare in the hands of those so deserving of confidence as the medical men. After all, our interests are the same. We are lieutenants under a captain, and our common foe is disease. It is to the doctor's interest to consider his subordinates, and ours to carry out his orders and respect his advice. Should the spirit to which I refer become too apparent in nurses, doctors, I believe, will train intelligent women to obey their orders, and trained nurses will not be required. If, on the other hand, the spirit of patience, self-forgenfulness, and tact, hand, the spirit of patience, self-forgetfulness, and tact, fostered and cultivated, the trained nurse, whose name sometimes inspires aversion to-day, would be again generally welcomed as the ministering angel.

### SHOULD FRIENDS BE ADMITTED AS PROBATIONERS?

"E. M. H." writes : I have read the pages of THE HOSPITAL with great interest for ten years, and I now venture to beg for a small space in the columns of your paper to ask the opinion of my professional sisters on a subject which may interest some of my fellow-workers—that of nurses' rights, or the consideration due to the feelings of a nurse. Two young girls, sisters, or intimate friends, who are refined, intellectual, sympathetic, in fact possessing all the qualities which we look for in the ideal nurse, apply to one of our large hospitals for admission. There are two vacancies, the candidates are in every way suitable, with one exception, they are friends. Should this friendship disqualify the candidates, and for what reasons? Nurses are expected to be bright and cheerful, always ready to sympathise with their suffering fellow-creatures. Can they do so when compelled by hospital restriction to give up all that is dear to them? Are there not cases where the weary nurse, to whom everything seems so difficult, may be encouraged to renewed efforts by the sympathy of the friend to whom she turns for consolation in her troubles, which at the outset seem so numerous? Her fellow-workers may, nay, generally are, kind to the poor "new pro.," though in a busy hospital ward there is little time to spare for our feelings; but the knowledge that she has a friend in the building would cheer the flagging spirits of the weary probationer. Many of our training schools will not admit candidates for the above-mentioned reason. It may be good for hospital discipline, but is it not rather hard on the nurse? In a hospital of three or five hundred beds would it not be possible to train these nurses without any ill effects to the discipline of the hospital? It is necessary for a nurse entering hospital to give up most of her former pleasures and friends, but in giving up all there is a possibility of the gentle, sympathetic woman developing into a mere machine, who lives only for self, and whose only interest is the quickest way she can obtain a position in her profession. I should be pleased to have the opinions of my professional sisters in this matter.

### Appointments.

BIRKENHEAD FEVER HOSPITAL.—Miss H. M. Perkins has been appointed charge nurse. She was trained at the Ingham Infirmary, South Shields, and has since been first assistant nurse at the North Eastern Fever Hospital, Tottenham.

CITY HOSPITAL, FAZAKERLY, LIVERPOOL.—Miss Edith Cutter has been appointed night superintendent. She was trained at Bethnal Green Infirmary, London, and the Sanatorium, Huddersfield. She has since been sister at Swallow's Nest Isolation Hospital, Sheffield; the Sanatorium, Huddersfield; and the City Hospital, East Liverpool.

COTON HILL HOSPITAL FOR THE INSANE, STAFFORD.—Miss Christina M. Fraser has been appointed matron. She was trained in general nursing at the Western Infirmary, Glasgow; received maternity training at the Glasgow Maternity Hospital; and holds the certificate of the Central Midwives Board. She has lately been assistant matron at the Stirling District Asylum, Larbert, N.B.

INFECTIOUS DISEASES HOSPITAL, BIRKDALE.—Miss Nora Beatrice Bannister has been appointed nurse matron. She was trained at Manchester Infirmary, and has since been ward sister, night sister, and assistant matron at Baguley Sanatorium, Timperley, Cheshire.

NORTHAMPTON GENERAL HOSPITAL.—Miss K. Hebdon has been appointed assistant matron and housekeeper. She was trained at Northampton General Hospital, and has since been sister and night superintendent.

OPEN-AIR SANATORIUM, WOKINGHAM.—Miss Florence Northfield has been appointed staff nurse. She was trained at St. Mary's (Islington) Infirmary, Highgate Hill, and has since been temporary staff and special nurse at St. Mary's Hospital, Paddington.

SHEFFIELD UNION INFIRMARY.—Miss Annie Henry has been appointed house sister, and Miss Emma Grace Brook and Miss Hannah Mary Carling ward sisters. Miss Henry was trained at Belfast Infirmary, where she afterwards became charge nurse. She has since been night superintendent at the Mater Infirmorum, Belfast, superintendent nurse at Bailieborough Infirmary, night nurse at Kilkenny Infirmary, night nurse at Carrickmacross Infirmary, and sister at Sheffield Union Infirmary. Miss Brook was trained at Sheffield Union Infirmary, where she afterwards became staff nurse. Miss Carling was trained at Middlesbrough Union Infirmary, where she was afterwards charge nurse.

THORPE INFECTIOUS DISEASES HOSPITAL.—Miss Eliza Knox Pinkney, has been appointed nurse matron. She was trained for maternity nursing at Newcastle-on-Tyne, and has been matron of Brandon Isolation Hospital, Brandon, County Durham.

### A Book and its Story.

### THE HOUSEHOLD AT HAWORTH.*

Mr. Clement Shorter's recent contribution to the "Literary Lives" series of "Charlotte Brontë and her Sisters" gives to the public in a collected form much fresh material, as well as some which has been published at different times since Mrs. Gaskell's "Life of Charlotte Brontë," but which has not been brought together under one cover until now. The author says justly in his introduction that Mrs. Gaskell wrote the only life of the gifted Charlotte that everyone should read. To the Haworth edition of that life Mr. Shorter was privileged to add many notes. In the present book he claims to disarm criticism by making the letters of Charlotte Brontë that have come to light since Mrs. Gaskell wrote tell their own story. Photographs are given of her father, the Reverend Patrick Brontë; of her husband, Mr. Nicholls; and a reproduction of the painting by Richmond of herself, with views of Haworth Vicarage and the house at Thornton, in Yorkshire, where she was born. So much has been written of the privations that Charlotte Brontë suffered in her home life that it comes as a welcome relief to read the following description of her at home, in the last years of her life at Haworth Vicarage, quoted by Mr. Shorter from Mrs. Gaskell's "Life": "I don't think that I ever saw a spot more exquisitely clean; the most dainty place for that I ever saw. No one comes to the house; nothing disturbs the deep repose; hardly a voice is heard; you catch the ticking of the clock in the kitchen or the buzzing of a fly in the parlour all over the house. Miss Brontë sits alone in her parlour, breakfasting with her father in his study at nine o'clock. . . . Then I accompanied her in her walks on the sweeping moors. Oh! those high, wild, desolate moors, up above the whole world and the very realms of silence. Home to dinner at two. Mr. Brontë has dinner sent in to him. All the small table arrangements had the same dainty simplicity about them. Then we rested, and talked over the clear bright fire; it is a cold country, and the fires gave a pretty dancing light all over the house. The parlour has evidently been refurnished within the last few years, since Miss Brontë's success has enabled her to have a little more money to spend. Everything fits into, and is in harmony with, the idea of a country parsonage possessed by people of very moderate means. The prevailing colour of the room is crimson, to make a warm setting for the cold grey landscape without; and two recesses on each side of the high, narrow, old-fashioned mantelpiece filled with books—books she has bought, and which tell the tale of her individual pursuits and tastes, not standard books." Not less attractive is the personal description of the author of "Jane Eyre," written about the same date, when the loss of her brother Branwell and her dearly loved sisters left her alone with her father, whose age and failing health were the cause of constant anxiety to her. This picture of Charlotte as she stands in the parlour of Haworth Vicarage is a vivid one. "I found Miss Brontë standing in the full light of the window, and I had ample opportunity of fixing her upon my memory, where her image is vividly present to this hour. She was diminutive in height and extremely fragile. Her hand was one of the smallest I ever grasped. She had no pretension to being considered beautiful, and was as far removed from being plain. . . . She had a most sweet smile, with a touch of melancholy in it. Altogether she was as unpretending, undemonstrative a little lady as you would well meet. Her age I took to be about five-and-thirty." So far the description does not suggest any of the striking characteristics which are associated with the strong creative faculty revealed in the author of "Jane Eyre." But the force of the further delineation of personality, as gathered from the exceptionally magnetic eyes of Charlotte Brontë, is both arresting and convincing. "When you saw and felt her eyes, the spirit that created Jane Eyre was revealed at once to you. They were rather small but of very peculiar colour, and had a strange lustre and intensity. They were chameleon-like, a blending of various brown and olive tints. But they looked you through and through, and you felt they were forming an opinion of you . . . by a subtle penetration into the very marrow of your mind and the innermost cores of your soul. . . . There was no boldness in the gaze, but an intense, direct, searching look, as of one who had the gift to read hidden mysteries and the right to read them. I had a feeling that I never experienced before or since, as though I had been mesmerised."

Of the three sisters Emily, Anne, and Charlotte, Emily is considered to be the greater genius. She died of consumption at the age of thirty in 1848. Her sister Anne succumbed to the same malady in the following year. Their brother Branwell died also in 1848. Charlotte, after attending them with self-sacrificing devotion, was left alone with her father, who was gradually going blind. She had not been without offers of marriage, which she had refused; and now, alone and bereft of her dear ones, she was approached by her father's curate, Mr. Arthur Bell Nicholls. Rumours had been circulated that she was going to marry Mr. Nicholls, and the following extract from her letter to a friend shows that she then had neither the wish to receive, nor the expectation of, an offer from him. Curates did not as a class appeal to Miss Brontë. "They seem to me," she says, "to be a self-seeking, vain, empty race," and of Mr. Nicholls she writes: "He and his fellow-curates regard me as an old maid, and I regard them one and all as highly uninteresting, narrow, and unattractive specimens of the coarser sex." But with an inconsistency peculiarly feminine she ultimately accepted Mr. Nicholls, in spite of strong opposition from her father, who hoped if his daughter married that she would find a husband who was more worthy of her acknowledged position in the literary world. Mr. Shorter's view of her ultimate acceptance of Mr. Nicholls is that it was due to the inherent love of justice characteristic of her sex. He says: "A woman hates injustice to a man who pays her the compliment of being in love with her, and she is nearly always in love with love. As a natural consequence, a few months found Charlotte Brontë deeply devoted to Mr. Nicholls," and the marriage very shortly afterwards took place. It is pathetic to read of the brief wedded happiness, which ended before twelve months were over in the death of Charlotte Nicholls. She wrote nothing of importance after her marriage, but these few months of married life were undoubtedly the happiest she had known. "We hear little of authorship, but they know little indeed of authorship," writes Mr. Shorter, "who think happiness in any robust sense and the writing of works of imagination are synonymous terms." The months Charlotte Brontë was writing her books were probably the most unhappy of her life. Now she took to domestic duties. "The married woman can call but a very small portion of each day her own," she writes. It is satisfactory to read that among her last letters was one written from her death-bed to a friend, in which she assures her of her husband's devotion to her. "I find in my husband the tenderest nurse, the kindest support, the best earthly comfort that ever woman had. His patience never fails, and it is tried by sad days and broken nights." And then come the most pathetic of "last words," surely as pathetic as any in the whole range of literary biography: "I am not going to die, am I? He will not separate us; we have been so happy." This further addition to Brontë memoirs will be welcomed by all Brontë lovers.

* "Charlotte Brontë and her Sisters." By Clement Shorter. (Hodder and Stoughton. 3s. 6d.)

### Notes and Queries.

**REGULATIONS.**

**The Editor is always willing to answer in this column, without any fee, all reasonable questions, as soon as possible.**

**But the following rules must be carefully observed.**

**1. Every communication must be accompanied by the name and address of the writer.**

**2. The question must always bear upon nursing, directly or indirectly.**

**If an answer is required by letter a fee of half-a-crown must be enclosed with the note containing the inquiry.**

*Various.*

(141) 1. Which is the quickest way to prepare chromic cut sutures? 2. Please tell me the address of the Up-country Association? 3. Also the address of the Nurses' Missionary Union?—*M. E. A.*

1. The question is not suitable for this column. 2. Hon. Secretary, Dalkeith House, Cambridge Park, Twickenham. 3. Write to the Hon. Secretary of the "Hospital Nurses' Union," Miss Dashwood, 5 Cambridge Gate, Regent's Park, N.W.

*Assistant Housekeeping.*

(142) Can you tell me if there are any appointments to be obtained in hospitals in the assistant-housekeeping departments?—*K. B. H.*

Yes; there are such appointments, but you can only find out about them by advertising, or by answering advertisements.

*Trained Nurses on Board Ship.*

(143) Will you kindly tell me if there are any passenger steamers that take a fully-trained nurse, and where to write to find out particulars?—*Max.*

There are only two lines of steamers at present employing trained nurses. For a situation as trained nurse combined with stewardess apply to the manager of the Royal Mail Steam Packet Company, Southampton, or as a nurse only, to the Booth Steamship Company, Liverpool; but vacancies are exceedingly rare.

*District Nurse.*

(144) Where can I get the names of the secretaries of the nursing associations for district work in the different counties? *L. M. G.*

If you mean Queen Victoria's Jubilee Institute nurses, you had better write to the General Superintendent, 120 Victoria Street, S.W.

*Colonial Nursing.*

(145) Will you kindly tell me to whom I should apply for particulars about the Colonial Nursing Service? Will you also tell me whether there is any nursing service for either Australia or Canada?—*Nurse B.*

Write to the Colonial Nursing Association, Imperial Institute, S.W. There is no special colonial service for either Australia or Canada. Both have plenty of nurses of their own.

*Nurse to Ship's Surgeon.*

(146) I am now a fully certificated nurse, having undergone three years' training. Can you give me advice how to become a ship's nurse?—*N. S.*

See reply to "*Max.*"

*District Nursing in Ontario.*

(147) How can I get an appointment as district nurse in Ontario? I have held an appointment here fore some years as district nurse, I have the best of testimonials and certificates from Queen Charlotte's Hospital, and hold the L.O.S. Can I practise as a midwife, or must I first get the certificate of the Central Midwives Board?—*Nurse M.*

You should think very seriously before you attempt to obtain an appointment as district nurse in a colony where good native nurses of all kinds are easy to obtain. Most certainly you must obtain the C.M.B. certificate. Then write to the Victorian Order of Nurses for Canada, 578 Somerset Street, Ottawa.

*Florence.*

(148) Are there any Nursing Institutes in Florence which employ English nurses?—*M. B.*

There is the Association of Trained Nurses and Masseuses, 7 Via Rondinelli, Florence.

**Handbooks for Nurses.**

| | Post Free. |
|---|---|
| "A Handbook for Nurses." (Dr. J. K. Watson.) ... | 5s. 4d. |
| "Nurses' Pronouncing Dictionary of Medical Terms" | 2s. 0d. |
| "Art of Massage." (Creighton Hale.) ... ... ... | 6s. 0d. |
| "Surgical Bandaging and Dressings." (Johnson Smith.) ... ... ... ... ... ... | 2s. 0d. |
| "Hints on Tropical Fevers." (Sister Pollard.) ... | 1s. 8d. |

Of all booksellers or of The Scientific Press, Limited, 28 & 29 Southampton Street, Strand, London, W.C.

### For Reading to the Sick.

SUPPLICATION.

Jesu, whelm'd in fears unknown,
With our evil left alone,
While no light from Heaven is shown,
Hear us, Holy Jesu.

When we seem in vain to pray,
And our hope seems far away,
In the darkness be our stay,
Hear us, Holy Jesu.

Though no Father seem to hear,
Though no light our spirits cheer,
May we know that God is near.
Hear us, Holy Jesu.

When the death-shades round us lour,
Guard us from the tempter's power,
Keep us in that trial hour.
Hear us, Holy Jesu.

No. 625 (*Hymns A. & M.*)

"My God, My God, why hast Thou forsaken Me?" In times of desolation and perplexity we ask this question. And surely we know the answer. It is to test our faith; and not only to test it, but to develop it. You ask me, perhaps, "How can I develop faith?" The answer is simple. By the practice of the much-neglected duty of meditation. It is in meditation that the Holy Spirit makes known to us the deep things of God.

The natural virtue of faith, on which all our business life depends, is a belief in human evidence—in a sense, a belief in one another. And yet how untrustworthy human evidence is; how often those in whom we put faith prove to be unworthy! How different is the supernatural virtue of faith, upon which our soul's life depends! This rests upon nothing less than God's truth, and gives us, as I have said, both strength and light; strength to climb to the mountain-top, from which we may gaze upon the land of promise, which is to be our home in eternity.

Though the darkness around me be so impenetrable that I cannot see one ray of light, yet will I trust Him, even more in adversity than in prosperity; for, "O God, Thou art my God." I do not ask to know why Thou dost treat me thus. Or, if I ask, I know I have already received the answer. It is enough for me that Thou art love, and can only deal with me in the spirit of love.

This life is not the end of all things for man. The Lord chasteneth those whom He loves, that they may be by discipline prepared for eternity.

*Rev. A. G. Mortimer, D.D.*

I could not do without Thee,
For years are fleeting fast,
And soon in solemn loneness
The river must be pass'd;
But Thou wilt never leave me,
And though the waves roll high,
I know Thou wilt be near me,
And whisper, "It is I."

*F. R. Havergal.*

